# Half a Century of Graft Survival After Deceased-Donor Kidney Transplantation: A Case Report

**DOI:** 10.1016/j.ekir.2023.03.006

**Published:** 2023-03-11

**Authors:** Daan Kremer, Stephan J.L. Bakker, Stefan P. Berger

**Affiliations:** 1 Division of Nephrology, Department of Internal Medicine, University of Groningen and University Medical Center Groningen, Groningen, The Netherlands

**To the Editor:**

In 1954, Joseph Murray performed a kidney transplantation between monozygotic twins, which is regarded as the first long-term successful kidney transplantation worldwide.^1^ The organ survived for 8 years. In 1962, the first successful deceased-donor kidney transplantation between genetically unrelated patients aided by immunosuppression allowed for significantly increased numbers of transplantations worldwide.^1^ In 1968, the first kidney transplantation in the University Hospital of Groningen, The Netherlands was performed.^2^ Three years later, this center’s 20th kidney was transplanted to the 30-year old female who is the subject of this report.

The patient had been on dialysis for 4 months, with biopsy-proven autosomal dominant Alport’s syndrome as the underlying kidney disease. She received a kidney from a 22-year old donor who died from traumatic injuries. The surgical procedure was successful. In the weeks after transplantation, kidney function improved. The patient was discharged 2 months after transplantation.

During the following years, kidney function remained stable, with azathioprine and prednisolone for immunosuppressive maintenance. Every additional 5 years of graft survival were considered a milestone, and were celebrated as such by the patient, who brought cake for the entire department. Treating physicians were struck by the sustained excellent kidney graft function (Figure 1). The patient survived more than 50 years after transplantation, relatively unbothered by her kidney disease. She recently passed away after a long and meaningful life. In the Supplementary Materials, the perspectives of the patient’s sister, as well as those of her former nephrologist and dialysis nurse (both retired for years) are described extensively. Notably, all of them mention the patient’s optimism, humor, and wittiness as the key to her graft survival.

**Figure 1:**
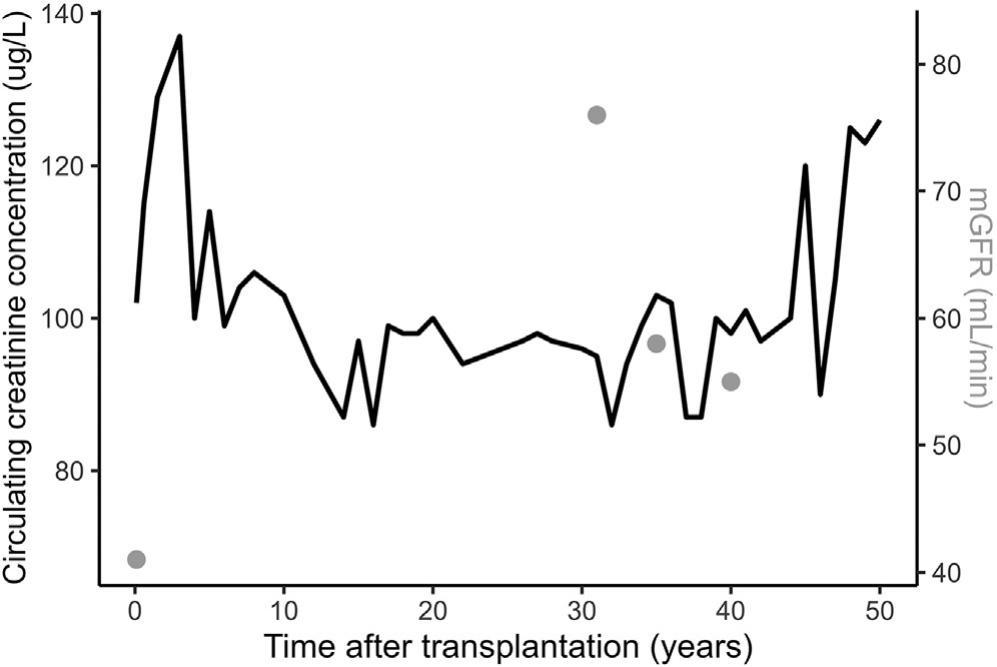
An overview of post-transplantation circulating creatinine concentration (left axis, line in black) and iothalamate measured glomerular filtration rate (right axis, dots in gray) over time. Notably, iothalamate-measured glomerular filtration rates were 76 ml/min and 55 ml/min at 30 and 40 years after transplantation, respectively. Moreover, creatinine levels remained rather stable, even up to 50 years after transplantation.

To our knowledge, this is the first report of a case with a deceased-donor kidney graft survival of over 50 years in the medical literature. This is absolutely remarkable, especially considering that kidney transplantation was still in its infancy in the early 1970s; reports from the late 1970s suggest 5-year graft survival rates <30%.^3^ All in all, this case of half a century of deceased-donor kidney graft survival is unique and shows the potential longevity of deceased-donor kidneys.

## Disclosure

All the authors declared no competing interests.

## Supplementary Material

Supplementary File (PDF)

Perspectives of the patient’s sister former treating nephrologist and dialysis nurse.
