# Supplementary material for: Half a Century of Graft Survival After Deceased-Donor Kidney Transplantation: A Case Report
Source: Kidney Int Rep. 2023 Mar 11;8(5):1123–4. doi: 10.1016/j.ekir.2023.03.006 (PMC10166725; doi:10.1016/j.ekir.2023.03.006)
Supplement: Supplementary File (PDF) [file mmc1.pdf]

Perspectives

Sister

The patient's sibling lovingly reminisces her sister: "Whoever got to meet my sister couldn't help but like her for her optimism, humor and wittiness." The patient's sister also suffered from Alport's syndrome and underwent a kidney transplantation, and both did everything in their power to maintain their kidney graft: "Like me, she did everything she could with regard to medication intake, hygiene and nutrition, as she felt those factors were absolutely essential to maintain her graft. For me, she has always been a perfect example of how one should deal with having a kidney disease, and receiving a kidney transplant."

Our patient had rough patches in her personal life, including the death of her son from complications after kidney transplantation. "However, she always kept her head up, and looked forward. She always did the best she could, gave everything, and made the best out of life, regardless of the situation she was in."

Caregivers

Both her former nephrologist and dialysis nurse have always kept in touch with our patient outside of the clinic. The described personality traits, endless optimism and the motivation to make the best out of life are also recognized by them. "Never in my career have I had a patient like her under my care", said our patient's former nephrologist (currently 88 years old, retired for over 20 years). "She had a very strong opinion about her own care, knew exactly what medication she was taking and why."

In the early 1970s, transplantation was still novel, and every patient eligible for transplantation was extremely happy to receive a call for a transplantation. Our patient has been very lucky to have received an excellent kidney from a young donor. "Indeed, it must have been an excellent donor kidney. However, I truly believe that it was her vibrant personality and optimism that kept her going,

and that made it possible to survive for more than 50 years with the same kidney”, says our patient’s former dialysis nurse.
